# Job vacancies at the European Centre for Disease Prevention and Control

**DOI:** 10.2807/1560-7917.ES.2017.22.12.30490

**Published:** 2017-03-23

**Authors:** Eurosurveillance editorial team

**Affiliations:** 1 European Centre for Disease Prevention and Control (ECDC), Stockholm, Sweden

The European Centre for Disease Prevention and Control (ECDC) invites applications for the following positions:

**Expert Data Management/ Knowledge Management**
**Expert Vaccine-preventable Diseases**

The deadlines for application are 18 April and 24 April 2017, respectively. For more information, visit the ECDC website: http://ecdc.europa.eu/en/aboutus/jobs/Pages/JobOpportunities.aspx

